# Developmental and Activity Dependent Regulation of Ionotropic Glutamate Receptors at Synapses

**DOI:** 10.1100/tsw.2002.74

**Published:** 2002-01-05

**Authors:** Elek Molnar, John T.R. Isaac

**Affiliations:** MRC Centre for Synaptic Plasticity, Department of Anatomy, University of Bristol, School of Medical Sciences, University Walk, Bristol BS8 1TD, UK

**Keywords:** AMPA receptors, glutamate, hippocampal neuron, kainate receptor, LTP, LTD, NMDA receptors, postsynaptic density, receptor targeting, silent synapses, synaptic development

## Abstract

Glutamate receptors mediate the majority of excitatory responses in the central nervous system. The establishment and refinement of glutamatergic synaptic connections depend on the concerted actions of a-amino-3-hydroxy-5-methyl-isoxazole-4-propionate (AMPA), *N*-methyl-D-aspartate (NMDA), and kainate (KA) type ionotropic glutamate receptors (iGluRs) and G-protein coupled metabotropic receptors. While a lot remains to be clarified, the most is known about the mechanisms by which the iGluR subtypes are targeted and how this is influenced by synaptic activity on both short and long time scales. Changes in their subunit compositions are also input specific and developmentally regulated. The identification of key molecular components of the postsynaptic density (PSD) and novel proteins that influence receptor targeting and clustering have started to reveal the underlying molecular mechanisms of the trafficking and targeting of iGluRs. Here we discuss the evidence that these basic mechanisms are used during developmental synaptic plasticity.

## INTRODUCTION

Synapses are modifiable sites of information transfer between neurons. This information transfer is mainly mediated by two neurotransmitters: glutamate and γ-amino-butyric acid (GABA). Glutamate, the major excitatory neurotransmitter, can activate several types of iGluRs, which are named for the agonists that selectively stimulate them: AMPA, NMDA, and KA[1,2,3]. iGluRs are formed from the presumed tetrameric or pentameric assembly of homologous subunits around a central ion pore[2,3]. The membrane topology of the iGluR subunits consists of a large extracellular amino-terminus, four membrane-associated domains with the second transmembrane domain (TM2) forming a re-entrant loop, and an intracellular C-terminus. NMDA receptors (NMDARs) are formed from hetero-oligomeric assemblies of NR1 subunits with NR2 (A–D) and NR3A subunits, whereas AMPA receptors (AMPARs) are built from subunits GluR1–4 and KA receptors (KARs) are formed from subunits GluR5–7 and KA-1 and 2 [2,3,4].

AMPARs are activated directly by glutamate binding, and their activation leads to changes in the membrane potential of the postsynaptic neuron. In contrast, NMDARs require the binding of glutamate and the coagonist glycine, as well as depolarisation, to become activated and conduct $Na^+$, $K^+$, and $Ca^{2+}$ ions. The voltage-dependent blockade of the NMDAR pore by $Mg^{2+}$ produces this additional requirement for depolarisation[5,6]. AMPARs are involved in the moment-to-moment information transfer between neurons, whereas one of the major roles of NMDARs is to act as detectors of specific patterns of activity that trigger long-term changes in synaptic strength by modulating AMPAR responses. Activation of NMDARs can be achieved by sustained activation of AMPARs at the same synapse, resulting in membrane depolarisation and relief of the voltage-dependent $Mg^{2+}$ block.

There is currently intense interest in the mechanisms regulating the functional properties and surface expression of iGluRs. In recent years an extensive effort has focused on the mechanisms regulating NMDARs and AMPARs. Although AMPARs and NMDARs are often located at the same postsynaptic sites, they are regulated in a differential manner. The phenomenon of silent synapses[7,8,9], a term used to describe glutamatergic synapses at which an NMDAR-mediated synaptic response can be evoked but which does not exhibit an AMPAR-mediated component, brought this differential regulation into sharp focus.

## GLUTAMATE TRANSMISSION AND SILENT SYNAPSES EARLY IN DEVELOPMENT

Recent studies indicate that glutamatergic synapses already operate in the late embryonic period. GluR1 and GluR2/3 AMPAR subunits have been detected by immunochemical methods at embryonic day 20 (E20) in rat CA1 pyramidal cell bodies[10,11]. This is in line with the identification of AMPARs at early embryonic stages (E14–15) in other rat brain regions including hypothalamic neurons[12] and raphe cells[13]. AMPA and NMDA receptor mRNAs have also been detected in CA1 early in development by *in situ* hybridization[14] and Northern analysis[15]. Glutamatergic synaptic transmission in the mammalian brain can be detected electrophysiologically at late prenatal stages and early in postnatal development — for example, in neocortex[16,17,18] and hippocampus[19,20]. Although synaptic responses mediated by both AMPARs and NMDARs are observed, it is clear that the majority of transmission at these early developmental periods is mediated by NMDARs. One hypothesis to explain this is that early in development the majority of synapses have NMDARs but no functional AMPARs. This is the so-called "silent synapse hypothesis"[7,9]. Evidence for silent glutamatergic synapses, although proposed in principle before, was first provided by experiments demonstrating a difference in the variability of NMDAR-mediated and AMPAR-mediated EPSCs at CA1 synapses in the hippocampus[7]. This was confirmed soon after by the direct observation that a proportion of CA1 synapses exhibited NMDAR-mediated EPSCs in the absence of an AMPAR-mediated component[8,9] (Fig. 1). In addition, it was shown in these studies that induction of NMDAR-dependent long-term potentiation (LTP) caused silent synapses to be rapidly converted into functional ones now expressing AMPAR-mediated responses.

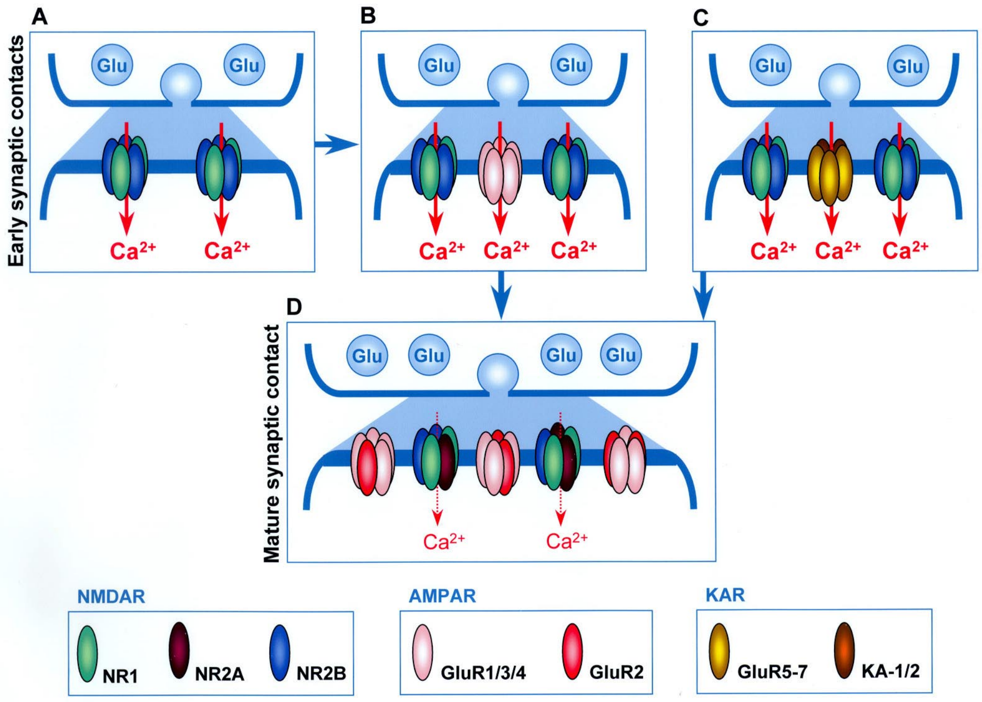

**FIGURE 1.** Schematic illustration of characteristic changes in the synaptic distribution and subunit composition of AMPA, NMDA, and KA receptors during development. Early in development, there are a large proportion of synapses containing only NMDARs (A) that subsequently acquire AMPARs to produce fully functional synapses (B and D). At hippocampal and intracortical synapses, once AMPARs are present there is a developmental increase in the relative ratio of GluR2 (B → D) compared to other AMPAR subunits on the neuronal surface. At cerebellar stelate cell synapses, a rapid incorporation of GluR2-containing AMPARs can be induced by synaptic activity. In hippocampal and neocortical neurons, early functional NMDARs are composed of combinations of NR1 and NR2B subunits (A). The NR2A subunit is gradually expressed from early postnatal ages and in the adult forebrain NMDARs are mainly composed of NR1/NR2A/NR2B heteromers. In thalamocortical synapses, KARs are exchanged with mature GluR2-containing AMPARs during development and in response to activity and NMDAR activation (C → D).

A key question that has been the subject of intense recent investigation concerns the mechanism underlying the electrophysiological observation of silent synapses and their conversion to a functional form during LTP. Currently there are three main competing hypotheses:

1. Postsynaptically silent synapses: This is the simplest and original explanation that silent synapses lack AMPARs at their postsynaptic membrane but do have NMDARs. During LTP AMPARs are rapidly inserted in response to NMDAR activation[7,8,9] (Fig. 1).
2. Spillover: Silent synapses express both AMPARs and NMDARs but have a release probability of zero and so are presynaptically silent. They sense glutamate spilling over from neighbouring functional synapses by virtue of the higher affinity NMDARs. LTP is due to an increase in probability of release[21].
3. Whispering synapses: Silent synapses have AMPARs and NMDARs but have an immature presynaptic release mechanism characterised by a slow release of glutamate at a low concentration. This is insufficient to activate postsynaptic AMPARs but does activate the higher affinity NMDARs. LTP is due to a switch in release of transmitter from the immature form to the fast mature mechanism, which is able to activate AMPARs[22,23].

The initial descriptions of silent synapses were from experiments performed on developing tissue (hippocampal slices from 4-week-old guinea pigs or from 2-week-old rats).

Subsequently it was shown that the incidence of silent synapses in the rat hippocampus was much greater earlier in development (e.g., 80% at postnatal day (P) 1–2), and that the numbers decreased with age during first postnatal week[19]. Around the same time a similar observation was also made in the optic tectum of *Xenopus* tadpoles; neurons in the less mature caudal part of the tectum had more silent synapses than those in the more mature rostral part[24]. Since these initial findings, silent synapses have been observed in many areas of the mammalian CNS, including the rodent barrel cortex[18], visual cortex[25], spinal cord[26,27,28], lateral geniculate nucleus[29] and dentate gyrus[30]. All these investigations were performed on developing tissue and a developmental down regulation of the number of silent synapses was reported in many of these studies[18,25,26,27,28,30].

One idea to explain this developmental down regulation is that specific patterns of synaptic activity during early development cause silent synapses to become functional. Indeed, since silent synapses can be converted to functional ones during LTP[7,8,9], one attractive hypothesis is that the developmental loss of silent synapses is due to a series of discrete LTP-like events in which activity converts individual synapses from silent to functional, thus producing this progressive decline in silent synapse number. For developing sensory pathways, a natural extension to this idea is that experience-driven activity producing LTP causes the progressive loss of silent synapses[18]. However, although these ideas have been around for a number of years, only recently have experiments been performed to start to address this issue, and things do not appear to be that simple.

The first indication that the link between activity and the developmental regulation of silent synapses may be more complex than originally thought is that if one compares the developmental profile for silent synapses in all the preparations in which it has so far been studied (hippocampus, spinal cord, and neocortex), it is noticeable that the profile is remarkably similar. In most preparations the number of silent synapses decreases to a low level by the end of the first postnatal week. If specific patterns of activity (perhaps driven by experience) were causing the developmental conversion of silent to functional synapses, one might expect that the profiles exhibit much greater differences between these structures. This rather uniform developmental profile may suggest that the conversion of silent to functional synapses is a general developmental mechanism in the brain and may not require specific patterns of activity to be expressed. Recent experiments, however, have gone further than this and suggest that neither synaptic activity nor NMDARs are required for the conversion of silent to functional synapses. In one study using hippocampal slice cultures, synapse development was studied in the continuous presence of TTX to block activity, and this did not block synapse development[31]. More surprisingly, however, NMDAR antagonists not only did not block synapse development but actually caused an excessive formation of functional synapses. Another recent study has also found that activity is not required for functional synapse formation[32]. Blockade of activity with TTX caused an increase in synaptic density and staining for the AMPAR subunit GluR1, and these changes were associated with an increase in the frequency of AMPAR-mediated miniature EPSCs.

These studies indicate that AMPARs can reach the surface of synapses in the absence of activity and NMDAR activation. This is clearly at odds with the idea that NMDAR-dependent LTP causes the unsilencing of silent synapses during development. One possibility to explain these conflicting results is that there are two processes regulating AMPAR surface expression at synapses during development: a global regulation of synaptic strength similar to the “synaptic scaling” phenomenon described for cultured neurons[33], and an input-specific activity-dependent LTP mechanism for the delivery and insertion of new AMPARs. Indeed, recent evidence suggests that this latter process occurs for GluR4-containing AMPARs[34]. Another possibility is that activity involving an NMDAR-dependent mechanism, rather than being important in driving AMPARs to the surface of silent synapses, may have an opposite role and serve to limit the surface expression of AMPARs at synapses[31]. One candidate process for this is NMDAR-dependent long-term depression (LTD). Recent work has shown that LTD involves the removal of AMPARs from the surface of cultured hippocampal neurons[35,36]. Furthermore, in hippocampal slices, LTD causes functional synapses to be converted to silent ones[37]. Therefore, a loss of NMDAR-dependent LTD could contribute to the excessive formation of functional synapses in the presence of NMDAR antagonists. Clearly the mechanisms for the developmental regulation of silent synapses are complex. Indeed, very recently it has been found that early in the first postnatal week neurons in layer II/III of the neocortex contain low numbers of silent synapses relative to the developmentally more mature layer VI neurons[38]. During development, layer II/III neurons initially show a developmental *increase* in the number of silent synapses before subsequently exhibiting the well-described developmental decrease. This suggests that there may be more than one developmental process regulating silent synapses and further investigations are required to investigate the interactions between these regulatory mechanisms.

## IMMUNOCYTOCHEMICAL ANALYSIS OF SYNAPTIC AMPA AND NMDA RECEPTOR DISTRIBUTION IN DEVELOPING NEURONS

Since the original reports of silent synapses, much effort has gone into investigating the anatomic substrate for the phenomenon, both in native brain tissue and in cell culture. Several immunocytochemical studies that used cultured hippocampal neurons have provided evidence for “morphologically silent” synapses that physically contain NMDARs but no AMPARs (reviewed in Craig and Boudin[39] and Molnar et al.[40]). Some glutamatergic synapses in culture lack AMPARs on their surface, but contain AMPARs within, or in close proximity to, their spines. Anatomical evidence for this includes the observation that cultured hippocampal neurons contain a high proportion of synapses that only express GluR1 intracellularly[41]. That these are not synapses that express GluR1-lacking AMPARs at their surface has been confirmed using an antibody, which reacts with all four AMPAR subunits (GluR1–4)[42]. Many of these synapses that lack surface AMPARs do express NMDARs at their surface and thus provide the anatomical substrate of “silent” synapses[43,44,45]. Recent studies demonstrated that newly formed excitatory synapses contain NMDARs and the clustering of AMPARs can be observed at NMDAR containing synapses as the neurons mature[44,45,46].

Antibodies against extracellular epitopes have allowed the direct colocalisation and quantification of surface-expressed native AMPA and NMDA receptors on cultured living hippocampal neurons during development[45]. Immunocytochemical analysis of hippocampal neurons between 3 and 20 days *in vitro* showed no change in the proportion of synapses expressing NMDARs (~60%) but a dramatic increase (~50%) in the proportion that also expressed AMPARs. Colocalisation with the presynaptic marker synaptophysin indicate that the majority of AMPA and NMDA receptors concentrate at synaptic sites on the cell surface and that there is a developmental decrease in the proportion of extrasynaptic clusters of both AMPA and NMDA receptors[45]. This redistribution to synaptic sites may be induced by the activity of newly formed synapses[47]. These results are consistent with electrophysiological data showing that the distribution of the pre- and postsynaptic terminals are rapidly synchronised and most synapses have NMDARs throughout development[48].

Parallel immunocytochemical and quantitative cell-ELISA experiments suggest an increase in both the number of AMPAR-containing synapses and the density of AMPARs at synapses during the first two postnatal weeks[45]. This is consistent with electrophysiological data showing that the developmental increase in AMPAR EPSCs is due to both an increase in synapse number and an increase in quantal size[49].

It is likely that similar arrangements exist in native tissue. Quantitative immunogold-labelling electronmicroscopic studies of CA1 neurons identified a large variability in the AMPAR content at individual synapses[50,51,52], suggesting that AMPAR number can be regulated over a wide range at native synapses. A large proportion of synapses contain very low or undetectable levels of AMPARs, whilst virtually all synapses on spines contain NMDARs[50,51,53]. Large synapses express the highest level of both receptors, whereas smaller synapses have a wide range of AMPAR content accompanied by a relatively uniform NMDAR content[53]. In small synapses, clusters of NMDARs predominate in the centre of the PSD and are accompanied by an even distribution of AMPARs when present, whereas in larger synapses, clusters of NMDARs are interspersed in a field of relatively evenly distributed AMPARs[54]. These observations suggest that the targeting mechanisms and the regulation of cell surface expression of AMPA and NMDA receptors follow different rules, which is consistent with results obtained using cultured hippocampal neurons.

Biochemical analysis of synaptosomal and microsomal fractions during development of the rat cortex has identified a developmental rearrangement in the distribution of AMPARs within neurons[55]. The synaptosomal/microsomal distribution of AMPARs changes from neonate to adult. In 2-day-old rats a large proportion of AMPARs were identified, mainly in the microsomal fraction, whilst in adult cerebral cortex the highest proportion of AMPARs are in the synaptosomal fraction. The microsomal AMPARs in developing neurons may represent a pool of intracellular receptors awaiting membrane insertion in response to appropriate cellular or developmental signals. This developmental rearrangement of the distribution of AMPARs within neurons is probably important in the processes of synaptic stabilisation and plasticity. NMDARs are not subject to the same developmental subcellular redistribution as AMPARs[55].

Low-density hippocampal neuronal cultures were used for immunocytochemical studies with subunit specific antibodies to analyse the time course of synaptic clustering of NMDA and AMPA receptors and PSD proteins[43,44,46,56]. When developing neurons establish connections, surface expressed iGluRs cluster at synapses. Clustering and retention of the receptors at postsynaptic sites appears to be dependent on intracellular anchoring proteins[57]. Some of these interacting PSD-associated proteins (e.g., PSD-95, chapsyn, GKAP[58,59]) redistribute during synaptogenesis following a pattern, which is consistent with a role in glutamatergic synapse formation[44,46,60,61].

NMDAR-dependent LTP has been observed in cultured hippocampal neurons using repetitive stimulation of the presynaptic partner of synaptically coupled neurons[62,63,64,65]. It has also been observed following field stimulation[66] or by the transient application of L-glutamate[67]. Recent studies provided direct morphological evidence that NMDAR activity can regulate rapidly the recruitment of native AMPARs into silent synapses[68,69,70] (Fig. 2). These studies used various procedures to induce LTP-like changes in cultured neurons, including (1) activation of NMDARs in cultured hippocampal neurons by elevating the concentration of the coagonist glycine to suprasaturating levels (100–200 µ*M*) for 3 min[69] or 15–20 min[68]; (2) spontaneous synaptic activity in low-density hippocampal cultures and high-density cortical cultures following the withdrawal of 200 µ*M* D,L-APV for 15 min[68]; and (3) transient depolarisation using a 3 × 1 s exposure to 90 m*M* KCl in postnatal dissociated hippocampal neurons[70,71] (Fig. 2). All these studies show that the activation of NMDARs results in the rapid recruitment of AMPARs at sites on the neuronal membrane that initially lack AMPARs within minutes. In parallel, an NMDAR-dependent increase in the frequency of AMPAR-mediated miniature EPSCs (mEPSCs) were found, indicating that the new “puncta” (Fig. 2) represented functional synapses[68,69,71].

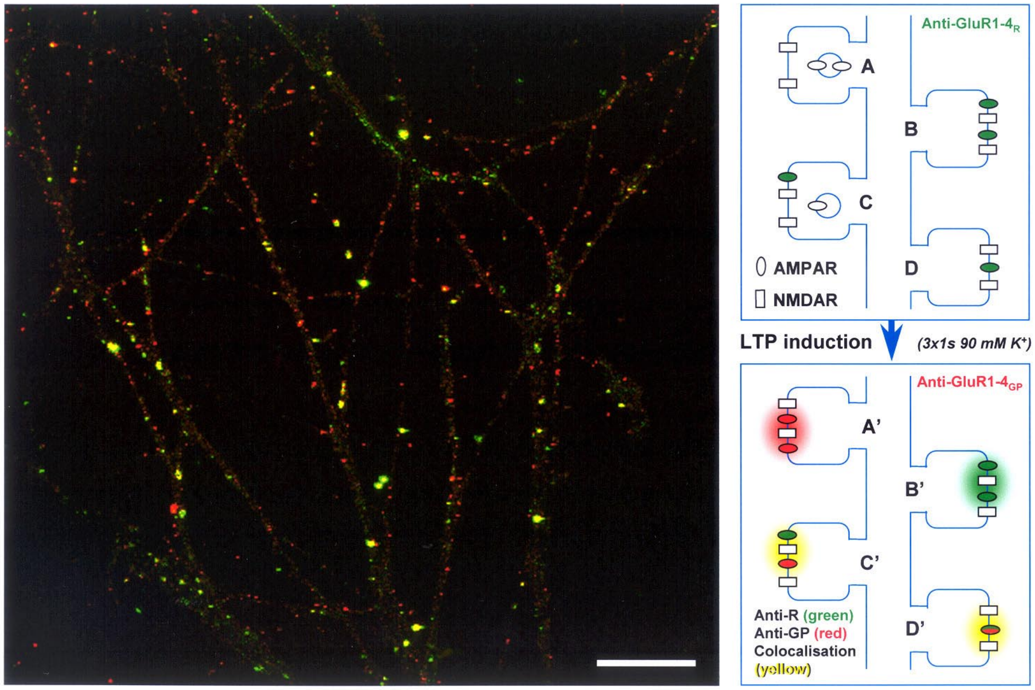

**FIGURE 2.** Changes in AMPAR expression on the surface of living hippocampal neurons following LTP induction. Site-directed antibodies that recognise an extracellular region of all AMPAR subunits (GluR1–4) were used for the immunolabelling of living neurons. These antibodies were raised in two species (rabbit: Anti-GluR1–$4_R$; and guinea pig: Anti-GluR1–$4_{GP}$) to enable sequential immunofluorescent labelling of individual living neurons before (green and yellow) and after (red) the induction of LTP by using $3 \times 1$ s exposure to 90 m*M* $K^+$. Sequential immunolabelling revealed the appearance of new AMPAR clusters (red) at sites on the neuronal surface that lacked AMPARs prior to LTP induction. Right panels schematically illustrate the colocalisation (yellow) of anti-GluR1–$4_R$ (green) and anti-GluR1–$4_{GP}$ (red) immunoreactivity on the surface of hippocampal neurons. In the lower right panel the possible results of an LTP experiment are illustrated: (A') newly surface-inserted receptors labelled only with the anti-GluR1–$4_{GP}$ following LTP induction (red); (B') receptors labelled prior to LTP induction with the anti-GluR1–$4_R$ antibody (green); (C') anti-GluR1–$4_R$ or anti-GluR1–$4_{GP}$ labelled receptors in close proximity (yellow).; and (D') previously anti-GluR1–$4_R$ labelled AMPARs that are relabelled with the anti-GluR1–$4_{GP}$ (yellow). The appearance of new AMPAR clusters was NMDAR-dependent, since it was antagonised by the application of NMDAR antagonists (not shown). Scale bar 20 μm. See[70,71] for detailed description of these experiments.

## CHANGES IN AMPAR SUBUNIT COMPOSITION DURING DEVELOPMENT

Changes in subunits comprising AMPA and NMDA receptor channels are important for long-term regulation of synaptic activity (Fig. 1). The early postnatal period is characterized by extensive changes in the expression of several iGluR subunits, implying developmental changes in the functional properties of the receptors[72]. The properties of AMPARs undergo developmental changes as a result of the differential expression of alternatively spliced and edited subunits. *In situ* hybridisation shows that *flip* forms of AMPAR subunits are expressed throughout embryonic and postnatal life and remain largely invariant during postnatal development. In contrast, *flop* forms arise at postnatal stages[14,73]. Receptors with *flip* sequences allow more current into cells than receptors containing *flop* sequences and this may be relevant to early-forming synapses.

The GluR2 subunit in the edited form is responsible for the $Ca^{2+}$ impermeability of AMPARs[3,72]. GluR2 subunit editing has been shown to have consequences for brain development. During embryonic development, a small number of receptors appear to have the

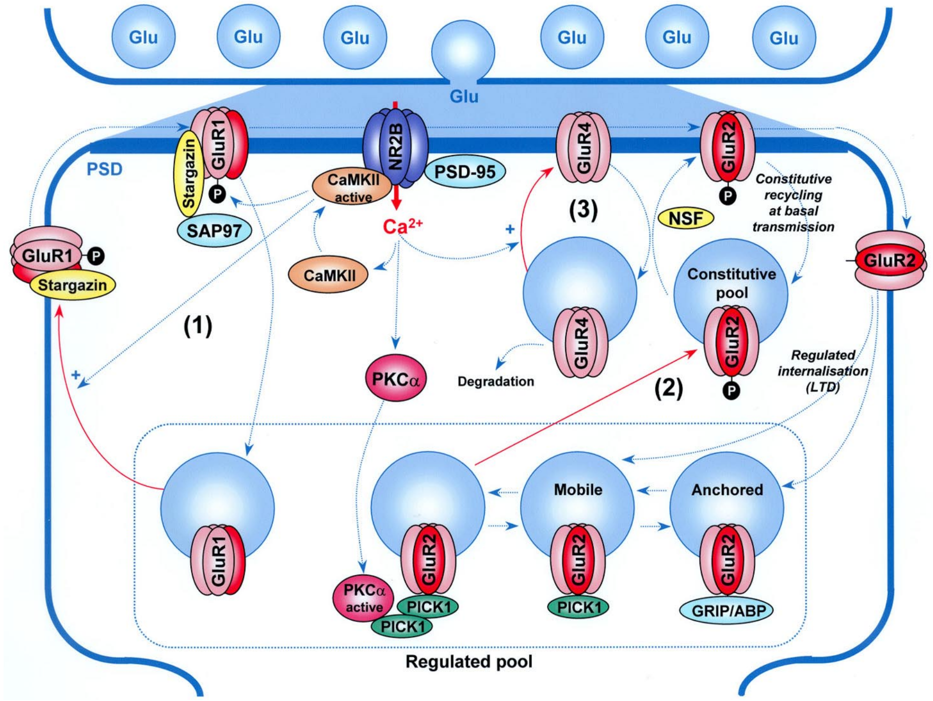

**FIGURE 3.** A schematic model for the differential regulation of AMPAR surface targeting by interacting proteins**.** Activation of NMDARs during LTP leads to insertion of AMPARs via at least three mechanisms (red arrows indicate NMDAR-regulated AMPAR insertion/translocation steps). NMDAR-dependent activation of (1) CaMKII causes insertion of GluR1-containing receptors involving the interaction with stargazing and SAP97 (synapse-associated protein 97), and (2) PKC causes insertion of GluR2-containing receptors involving the PDZ interactions with C-terminal GluR2. In addition, (3) GluR4-containing receptors are inserted at silent synapses early in development in response to NMDAR activation but not requiring CaMKII and then are exchanged for GluR2. GluR1-containing receptors are also functionally upregulated by CaMKII-dependent phosphorylation during LTP. NMDAR activation induces the translocation of CaMKII to the synapse, where it can directly interact with the NR2B NMDAR subunit, which locks CaMKII in an active conformation. GluR2-containing receptors are constitutively recycled involving the NSF-GluR2 interaction. NMDAR-dependent LTD dephosphorylation of GluR2 leads to the internalisation of AMPARs via dynamin/clathrin-mediated endocytosis. The internalised receptors enter the regulated pool where their reinsertion is prevented by their binding to GRIP. NMDAR-dependent activation of PKC involving PICK1 is necessary for these receptors to be reinserted.

unedited GluR2 subunit; but with postnatal development, virtually all GluR2 subunits are edited[72]. There is a developmental increase in the relative ratio of GluR2 compared to other AMPAR subunits on the neuronal surface (Fig. 1). The proportion of AMPAR positive synapses expressing the GluR2 subunit show an increase from ~67% to ~96% during the first two postnatal weeks [45]. The relative increase in synaptic GluR2 can reduce $Ca^{2+}$ influx by forming $Ca^{2+}$-impermeable AMPARs. The relative increase in synaptic GluR2 continues during the second week, whereas there are only moderate changes in the surface expression and total amount of AMPARs, suggesting that this process is independently regulated[45]. These results indicate that not only the expression and synaptic targeting of AMPARs are regulated, but that the subunit composition is also under developmental control. These results are consistent with electrophysiological studies in brain slices reporting changes in $Ca^{2+}$-permeability consistent with the incorporation of GluR2 subunit-containing AMPARs during development in neocortex[74] and the activity-dependent insertion of $Ca^{2+}$-impermeable AMPARs at cerebellar stellate cell synapses[75].

A recently developed approach using hippocampal organotypic slice cultures combined with viral infection of neurons with GFP-tagged receptor subunits has provided novel insight into the mechanisms for the insertion of AMPARs at synapses[76,77] (Fig. 3). NMDAR-dependent LTP involving a calcium/calmodulin-dependent protein kinase II (CaMKII) mechanism is required for the insertion of GluR1 homomers[76,78,79], while GluR2 homomers appear to be inserted in a constitutive and activity-independent manner at already functional synapses[34,79]. An important feature of this approach is that slice culture is a developing tissue, making this technique particularly useful in the study of the developmental regulation of receptor subunit trafficking and insertion. Using this approach, the same group found that GluR4 homomers are preferentially inserted into silent synapses at P5–7 in an activity- and NMDAR-dependent manner and then are gradually exchanged for GluR2-containing receptors in an activity-independent process that maintained synaptic strength[34]. The insertion of GluR4 was not dependent upon αCaMKII and no other subunit was found to be inserted into silent synapses. This seems a good candidate mechanism for developmental LTP. It has many of the features of developmental LTP already described from other investigations as discussed below: it is GluR1 independent, it involves an insertion of receptors and the unsilencing of silent synapses, and it does not require αCaMKII. Although much progress has been made in understanding the regulation and trafficking of AMPARs using slice culture and viral delivery of GFP-tagged subunits, this technique does, however, suffer from the disadvantage that infected neurons are overloaded by the virally expressed subunit. This means that an excess of homomeric AMPAR complexes are synthesised, which is likely to have effects on the regulation of trafficking and insertion. This is demonstrated by the fact that homomeric receptor complexes are expressed at the surface, which is not observed for endogenous receptors in neurons. Although some progress has been made in this direction by coexpressing two subunits[79], this drawback of the technique means that further investigations are needed to understand the regulation of the native heteromeric receptor subunits.

## DEVELOPMENTAL CHANGES IN NMDAR SUBUNIT COMPOSITION

NMDARs are present at many glutamatergic excitatory synapses in the CNS and display unique properties that depend on their subunit composition; (1) the requirement for the coagonist glycine for activation; (2) voltage-sensitive block by $Mg^{2+}$; (3) high permeability to $Ca^{2+}$; and (4) slow activation/deactivation kinetics. Several distinct NMDAR subtypes have been identified in the CNS, differing in their sensitivity to endogenous and exogenous ligands, permeation and block by divalent ions, kinetic properties, and interaction with intracellular proteins[80,81]. The subunit composition of NMDARs is under developmental control. While *in situ* hybridisation and single-cell polymerase chain reaction studies have shown that that the NR1 subunit is ubiquitously present at a high level in virtually all rat brain regions at both embryonic and postnatal ages, the mRNAs for NR2A–D subunits are differentially distributed throughout the brain, with patterns of expression that change strikingly during development[82,83]. At birth, forebrain NMDARs are composed almost exclusively of NR1 and NR2B subunits, gradually incorporating more NR2A subunits during postnatal development. This developmental decrease in the forebrain NR2B:NR2A ratio is complete by the third or fourth postnatal week in rodents. In the embryonic brain, the NR2D subunit is present in the diencephalon and brainstem. Soon after birth, NR2A mRNA is found in most regions, whereas NR2C appears later and is only prominent in the cerebellum[82,83]. The stoichiometry of native NMDARs in the plasma membrane is not yet known; both tetrameric[84] and pentameric models have been proposed[85]. In the adult hippocampus and neocortex, NMDARs are mainly composed of NR1/NR2A/NR2B heteromers[85,86], but the presence of other subunit combinations (e.g., homo-oligomeric complexes of NR1) have also been proposed[87]. Functional studies have examined the possible subunit composition of NMDARs in a number of identified neurons in various regions of the CNS[81]. There appears to be a general trend towards a decreasing (but still significant) contribution from the NR2B subunit during development, which is associated with an increased contribution of NR2A-containing NMDARs to the synaptic current. These electrophysiological studies have reported that the duration of NMDAR-mediated synaptic responses is shorter in older animals compared to younger ones. These changes correlate with the developmental switch in expression of NR2B subunit with slower deactivation kinetics to the NR2A subunit, which imparts faster deactivation kinetics[81]. Synaptic activity is a key factor in the regulation of NR2A and NR1b NMDAR subunit expression during development. A rapid and long-lasting change in subunit composition has been identified for NMDARs in response to synaptic activity[88,89] and experience[88], which suggests differential expression and synaptic targeting of various subunit combinations. While earlier reports established that NMDARs do not cycle rapidly in and out of the postsynaptic membrane under normal conditions, it is likely that the surface expression of NMDARs is also tightly regulated. However, new reports indicate activity-regulated rapid synaptic delivery and subunit specific internalisation of NMDARs[90,91,92,93]. The switch in the NMDAR subunit composition seems to depend on intracellular transduction pathways mediated by the cell adhesion molecules integrins[94]. The changes in the kinetic properties and $Ca^{2+}$ permeability of these receptors are thought to be critical in the role that NMDARs play in many activity-dependent developmental processes.

In a recent study, the above-described developmental change to more NR2A subunits was reversed using transgenic mice overexpressing the NR2B subunit[95]. In the adult NR2B transgenic mice glutamate evoked larger and longer NMDAR-mediated currents and LTP was greatly enhanced (which was reminiscent of LTP in young rather than adult animals) probably due to the enhanced $Ca^{2+}$ flux. The NR2B transgenic mice also showed improved learning scores compared with normal mice in different tests of their ability to acquire and retain information[95]. These experiments suggest that the NMDARs serve as a graded molecular switch for gating the age-dependent threshold for synaptic plasticity and memory formation.

Another interesting observation is that susceptibility to LTP at thalamocortical synapses in early postnatal mouse slices was lost at a time point when the duration of NMDAR-mediated EPSCs was not significantly altered. This suggests that dramatic changes in the kinetics of NMDAR EPSCs are not necessarily required for changes in synaptic plasticity mechanisms. However, changes in the subunit composition of NMDARs (as defined pharmacologically using ifenprodil) correlate strongly with the loss of the ability to generate LTP[96]. The strong correlation between the ifenprodil sensitivity of NMDAR EPSCs and the critical period for LTP suggests that the subunit composition of NMDARs at thalamocortical synapses may influence their susceptibility to LTP. It is possible that the loss of NR2B at NMDARs, accompanied by the addition of other NMDAR subunits, contributes to the modification of signal transduction cascades that are coupled to NMDAR activation[97,98], and this in turn is responsible for the loss of LTP. A recent study reported that regulated αCaMKII interaction with NR2B provides a mechanism for the glutamate-induced translocation of the kinase to the synapse[99]. αCaMKII is critically important for synaptic plasticity (reviewed in Soderling and Derkach[100]). The interaction with NR2B can facilitate αCaMKII response to synaptic $Ca^{2+}$, and locks the kinase in an active conformation[99]. This direct activation of CaMKII by its interaction with the NR2B can provide one possible explanation of the developmental loss of LTP in the thalamocortical synapses[96] where the NMDAR subunit exchange process at the synaptic level could be different from hippocampal neurons where LTP is maintained.

## DEVELOPMENTAL CHANGES IN KAR

Although the developmental role for KARs is not clear, the expression of KAR subunit transcripts and their mRNA editing change markedly during development. For example, GluR5 gene expression peaks around birth in the sensory cortex, in CA1 hippocampal interneurons in the stratum oriens, in the septum, and in the thalamus. GluR6 shows a prenatal expression peak in the cingulated gyrus of the neucortex. KA-1 transcripts appear with the development of the hippocampus and remain largely confined to discrete areas such as the CA3 region, the dentate gyrus, and subiculum. KA-2 transcripts are found throughout the CNS from as early as E12 and remain constant until adulthood[101]. Single-cell reverse transcriptase polymerase chain reaction

(RT-PCR) has shown that only GluR6 subunit mRNA is present in most of the cultured embryonic hippocampal neurons exhibiting the characteristic native KAR response[102]. Cerebellar granule cells contain functional KARs both *in situ* and in culture, and RNA editing of GluR5 and GluR6 increases and KA-2 expression decreases as granule cells mature[103]. Editing of the Q/R site in GluR5 and GluR6 mRNAs occurs at very low levels in embryonic brain and increases to approximately 50% (GluR5) and 80% (GluR6) of the mRNA transcripts within the first few days after birth in most regions of the brain[104]. This developmentally regulated editing alters a critical residue producing subunits, which combine to form receptors with much lower $Ca^{2+}$ permeabilities and single-channel conductances. Changes in KAR $Ca^{2+}$ permeability could be involved in the regulation of synapse formation, stabilisation and plasticity[2,105].

A recent study has shown that functional KARs at thalamocortical synapses in the developing rodent barrel cortex are subject to developmental and activity-dependent regulation[106]. With a developmental profile that correlates with the critical period for experience-dependent plasticity at this input, the contribution of KARs decreases, an effect also produced acutely by the induction of NMDAR-dependent LTP. This suggests that KARs are regulated in response to synaptic plasticity during development. Interestingly, the synaptic KARs at this input exhibit a strongly rectifying current-voltage relationship, suggesting that they are permeable to $Ca^{2+}$, although the functional consequences of this are unknown. Further studies are required to understand the functional consequences this switch from slow KAR- to fast AMPAR-mediated transmission has for synaptic transmission and sensory processing in the barrel cortex.

## DEVELOPMENTAL CHANGES IN LTP EXPRESSION MECHANISMS

Models of synaptic plasticity, such as LTP and LTD of glutamatergic synapses, are believed to underlie the cellular basis of learning and memory in the adult brain and the activity-dependent regulation of synapse formation in developing brain[107,108,109,110]. The mechanisms involved in the induction and expression of LTP are likely to be critically involved in the synaptic modifications underlying learning and memory[107,110]. Two forms of LTP in hippocampal slices have been distinguished on the basis of whether their induction requires the synaptic activation of NMDARs or not. Most interest has focused on NMDAR-dependent LTP, which seems to be the predominant form in the vertebrate CNS, both *in vivo* and *in vitro*. The role of NMDARs in the induction of LTP is well established[107,111] and it is known that a rise in postsynaptic $Ca^{2+}$ is also essential for the induction of LTP[112]. Less is known about the signalling mechanisms that transduce the NMDAR-initiated $Ca^{2+}$ transient into a persistent change in synaptic strength. Current evidence suggests that activity-dependent changes in synaptic strength, such as LTP and LTD, result at least in part from changes in AMPAR-mediated responses. These changes may be attributed to altered levels of AMPARs in the postsynaptic membrane, altered receptor properties, or a combination of these two mechanisms. It has been reported previously that AMPARs are phosphorylated and dephosphorylated during LTP and LTD, respectively, and that this may lead to changes in the ion channel conductance or open channel probability of AMPARs during these processes (reviewed in Soderling and Derkach[100] and Malenka and Nicoll[110]). Numerous recent studies also suggest that the number of synaptic AMPARs also can be modulated by activity-dependent mechanisms[77,113]. Therefore at least two distinct postsynaptic mechanisms exist for the regulation of AMPAR function during synaptic plasticity.

The study of the expression mechanisms of NMDAR-dependent LTP is a highly controversial field, with many laboratories providing apparently conflicting results. However, many of these different and seemingly conflicting studies have used hippocampal slices obtained from animals of different ages. In particular, results from young and adult animals have been compared. With the advent of the whole-cell patch-clamp technique, adolescent rat hippocampus (from 2- to 4-week-old animals) has become the preparation of choice because of the greater ease of obtaining recordings. These patch-clamp studies have provided some of the most detailed investigations into LTP expression mechanisms and have presented evidence for a number of different processes underlying both LTP and LTD. One of the reasons for this is that adolescent hippocampus may still be undergoing rapid developmental changes in synaptic plasticity mechanisms and small age differences between studies (typically a difference of 1 week or less) could be highly significant in terms of which mechanism most prominent. All these factors could contribute to the apparent conflict in results between different studies. Therefore, to fully understand the mechanisms of LTP and LTD and the role of these processes in development and adult learning and memory, it is important to investigate the developmental regulation of these processes.

Some of the earliest evidence that LTP expression may be developmentally regulated came from the finding that silent synapses could be converted to functional ones during LTP induction[7,8,9]. This finding, together with the observed developmental down-regulation of silent synapses in hippocampus[19], suggests that unsilencing may be a developmental form of LTP. Further evidence in support of this idea is provided by studies demonstrating that NMDAR-dependent LTD, which is associated with the removal of AMPARs from synapses[35,36] and the resilencing of synapses[37], is only readily observed in developing (1- to 3-week-old) hippocampus[114,115]. Around the same time that LTP at silent synapses was described, it was also reported that a presynaptic mechanism for LTP was developmentally regulated[116]. In this study, it was found that the probability of release (Pr) at CA1 synapses during the first postnatal week was high, that LTP could not be induced at this age, and that during the second week of life Pr decreased coinciding with the appearance of LTP. This was used as evidence for an increase in Pr as the expression mechanism for LTP. This view now seems almost certainly to be incorrect since a number of laboratories have reported that LTP does occur in the first postnatal week at these synapses[19,22,117]. However, there is good evidence that there is an important switch in the postsynaptic signalling mechanisms downstream of NMDAR activation at this age. The expression of αCaMKII, an enzyme generally thought to be critical for the expression of adult LTP[100], is developmentally regulated. αCaMKII expression is low in the cortex in the first postnatal week but increases rapidly during the second week of life[118]. Evidence for a concomitant switch in the requirement for αCaMKII in LTP (and LTD) is provided by knock-out studies which show that plasticity is present in hippocampus and sensory cortex of young αCaMKII knock-out mice[119,120] but is absent in adults[121]. Thus, LTP during the first postnatal week is likely to involve other CaMKII isoforms[122] or other kinases such as PKC[110].

There is also accumulating evidence for multiple coexisting expression mechanisms for LTP during the second postnatal week, which may well represent the coexistence of adult and neonatal forms of LTP. A number of studies have reported more than one distinct mechanism existing simultaneously within the same set of experiments. For example, failures analysis of LTP at CA1 synapses in hippocampus in 14- to 18-day-old reveals two populations of synapses, in one of which LTP is associated with an increase in quantal amplitude in the absence of any failure rate change suggesting an increase in existing AMPAR function, and the other involving failure rate changes suggestive of the insertion of new AMPARs at silent synapses[123]. More recently it was found that LTP at P12–15 was associated with an increase in the single-channel conductance of AMPARs in two thirds of neurons, but in the remaining one third a similarly robust LTP was associated with no change in channel conductance but rather with an increase in the number of activated receptors[124]. More work needs to be carried out to determine if these coexisting mechanisms are indeed differentially developmentally regulated. However, recently strong genetic evidence has emerged for developmental regulation of LTP expression mechanisms. Two recent studies have investigated synaptic transmission and plasticity in a GluR1 knock-out mouse[125,126]. Surprisingly there were no electrophysiologically detectable differences in basal synaptic transmission at CA1 synapses in adult animals although LTP was absent. In addition, LTP was normal in 2-week-old animals[127]. Together this suggests that at least two forms of LTP exist: a GluR1-independent form involved in development, and a GluR1-dependent form that underlies adult LTP. This analysis of GluR1 knock-out mice, together with other recent studies of the molecular mechanisms of regulating AMPARs during synaptic plasticity[77,100,110,113,128,129] suggests that adult LTP may involve the phosphorylation and/or insertion of the GluR1 subunit of AMPARs at already functional synapses, while neonatal LTP involves the rapid insertion of AMPARs into silent synapses involving protein-protein interactions with other subunits (Fig. 3).

One disadvantage with the use of brain slices to study expression mechanisms of LTP is that complimentary techniques such as immunocytochemistry and imaging cannot be readily used. This restricts the analysis in brain slices to electrophysiology, the results of which are always open to multiple interpretations. Thus the use of techniques such as primary culture and organotypic culture together with a number of cell biological and imaging techniques, although not without their own disadvantages, has provided some novel insights into the expression mechanisms of synaptic plasticity. Evidence from these studies also points to multiple expression mechanisms. Early evidence supported an increase in probability of glutamate release due to observed increases neurotransmitter vesicular recycling[130]. However, recent studies show that NMDAR-dependent LTP causes an insertion of AMPARs both at silent and existing functional synapses[68,69,70,71]. This may once again reflect developmental regulation of expression mechanisms, since neuronal culture clearly is a developing system. Comparison of data from different investigations using primary culture is especially difficult, since small differences in culture conditions may have significant effects on the rate of development and the expression mechanism that predominates. A further confounding issue with primary culture is that some laboratories use postnatal neurons and other embryonic neurons, which may have consequences for the plasticity mechanisms expressed.

## INTERACTION PARTNERS ARE INVOLVED IN THE TRAFFICKING AND TARGETING OF iGluRs DURING DEVELOPMENT AND PLASTICITY

The molecular mechanisms regulating the differential targeting and clustering of various AMPA and NMDA receptors remain largely unknown, but proteins that interact directly or indirectly with AMPA and NMDAR subunits are likely to play central roles in this process[57,58,59,79,98,131,132,133] (Fig. 3).

Of the iGluR-interacting proteins, the best-described role in development is that for PSD-95 (postsynaptic density protein of 95 kDa). PSD-95 is a membrane-associated guanylate kinase (MAGUK) that contains three PDZ domains and is central to the formation of the NMDAR-associated multiprotein complex (reviewed in Garner et al.[58], Grand and O'Dell[97], Kennedy[134], and Sheng and Pak[135]). Overexpression of PSD-95 in hippocampal neurons can promote the maturation of excitatory synapses, as evidenced by enhanced synaptic clustering and function of NMDA and AMPA receptors[57]. Interestingly, PSD-95 overexpression also mediates a retrograde signal to enhance the maturation and function of presynaptic nerve terminals. This retrograde signal may be mediated via the neuroligin-β-neurexin interaction[136]. Targeted disruption of PSD-95 in mice has not produced abnormalities in synaptic structure[137]. The normal neuronal development in mice with mutant PSD-95 could be due to molecular redundancy, as four additional membrane-associated MAGUKs are expressed at the PSD of neurons. Alternatively, the normal receptor expression could be due to the fact that NMDARs interact with several additional PDZ domain-containing proteins. The overexpression of the PSD-95 binding protein Shank also promotes pre- and postsynaptic development in a manner that is enhanced by homer, a protein that interacts with mGluRs[138].

One prominent recent hypothesis is that AMPARs are rapidly recycled at the postsynaptic membrane and the regulation of this mechanism is utilised as an expression mechanism for LTP and LTD[37,113,139,140]. The first evidence for recycling of AMPARs at the postsynaptic membrane came from studies that used peptide inhibitors to disrupt the interaction between C-terminal GluR2 and the fusion protein *N*-ethylmaleimide-sensitive factor (NSF)[139,141,142]. This manipulation depresses AMPAR-mediated EPSCs[139,142], suggesting that the NSF-GluR2 interaction is important in the maintenance of surface-expressed synaptic AMPARs. This idea was further supported by the finding that antibody staining for surface-expressed AMPARs in living cultured neurons was greatly reduced when the NSF-GluR2 interaction was blocked[42]. Blockade of the NSF-GluR2 interaction also occludes NMDAR-dependent LTD[37,140], suggesting that the rapid recycling of AMPARs is also involved in the expression of LTD. Again, further evidence for this was provided from immunocytochemistry experiments in culture showing that LTD is associated with the removal of surface-expressed AMPARs from synapses[35,36] involving a dynamin/clathrin dependent process[143].

The extreme C-terminus of GluR2 binds to a pair of multi-PDZ domain-containing proteins (glutamate-receptor-interacting protein; GRIP and AMPA-receptor-binding protein; ABP) and also to a single PDZ protein PICK1 (protein interacting with C kinase 1)[131]. Interestingly, phosphorylation of the GluR2 PDZ-binding site by PKC selectively disrupts binding of GluR2 to GRIP/ABP, but leaves PICK1 binding intact[144]. Disruption of the PDZ-interacting proteins potentiates basal synaptic transmission in a PKC-dependent manner by producing an upregulation of AMPAR function at silent synapses in 2- to 3-week-old hippocampus[145,146]. This interaction is important for the expression of synaptic plasticity since its disruption prevents 5-HT induced potentiation in young (P4-21) spinal cord dorsal horn neurons[147] and blocks NMDAR-dependent LTD in hippocampus[145,146]. Interestingly, Daw et al. reported that the potentiating effects on basal transmission of blocking the PDZ interactions at CA1 synapses were only observed in one third of cells[145]. This one third–two thirds split in the mechanism regulating synaptic AMPARs is reminiscent of the ratio of the two mechanisms of expression for LTP obtained from conductance analysis of EPSCs using dendritic recordings from CA1 cells in slices obtained from animals of the same age[124]. This, together with the findings that (1) blockade of the PDZ-GluR2 interactions causes a selective increase in AMPAR function at silent synapses, (2) both the NSF and PDZ interactions with GluR2 are important for a developmental form of synaptic plasticity (i.e., NMDAR-dependent LTD), and (3) GluR2 is selectively incorporated into AMPAR complexes during development[45,74] and in response to activity[75], suggests that these C-terminal GluR2 interactions may be important for the developmental regulation of AMPAR function. Recent studies using virally overexpressed GFP-tagged AMPAR subunits in hippocampal slice cultures[77] lend some support to this idea. GluR2 subunits are exchanged for GluR4 subunits early in development[34]. GluR2 was not found to be inserted at silent synapses, but rather GluR1 was instead. Thus further investigations into the role of GluR2-interacting proteins in development both in slice culture and acute slices are warranted to elucidate the precise roles in developmental plasticity for these and other interactions (Fig. 3).

Two other AMPAR-interacting proteins were recently identified that may have a role in development. Stargazin is a transmembrane protein, which interacts with AMPARs[148]. The cytoplasmic C-terminal domain of stargazin contains a PDZ-binding site, which binds to PSD-95 and related proteins. The transmembrane domains of stargazin interact with AMPARs and regulate the delivery of these receptors to the cell surface (Fig. 3). Following insertion to the plasma membrane, the PDZ-binding site of stargazin interacts with PDZ domain containing scaffolding proteins (e.g., SAP-97) and targets the AMPARs to synapses. Narp is a member of pentaxin family of secreted lectins enriched at excitatory synapses and produced from both pre- and postsynaptic sources. Narp can facilitate the formation of new excitatory synapses and is able to cluster AMPARs at established synapses by forming oligomers[149]. Narp is regulated by synaptic activity and its dynamic expression provides a possible mechanism for activity-dependent synaptogenesis and synaptic plasticity. Whilst there is no further information available about the role of stargazin and narp in development, they probably play an important role.

## CONCLUSIONS

Evidence from several studies indicates that LTP and early postnatal development are mechanistically related and addition of AMPARs to the postsynaptic density is likely to play an important role in changes of synaptic efficacy. AMPARs residing on the cell surface are in dynamic equilibrium with those residing in intracellular transport vesicles. The rate of endocytosis and exocytosis of AMPARs is regulated by activity. A net increase in endocytotic rate will result in a decreased response size and a net increase in exocytotic rate will have the opposite effect. Further research into the cellular pathways involved in the differential targeting of AMPA, NMDA, and KA receptors will lead to a better understanding of the role of iGluRs in neuronal development, memory, learning, and disorders affecting the nervous system.

## ACKNOWLEDGEMENTS

This work was supported by the Medical Research Council and the Wellcome Trust. We thank the Medical Research Council for providing an Infrastructure Award to establish the School of Medical Sciences Cell Imaging Facility.

## BIOSKETCH

**Elek Molnar**, Ph.D., is a Reader in Neuroscience at the MRC Centre for Synaptic Plasticity, University of Bristol, School of Medical Sciences, Bristol, U.K. His reseach interests include mechanism of ionotropic glutamate receptor (iGluR) assembly, cell surface targeting, synaptic expression and the role of receptor-associated proteins; the role of different iGluRs in long-term activity-dependent changes in the strength of synaptic transmission; immunocytochemical and biochemical analysis of the cellular and subcellular visualization of various native iGluR subunit proteins in cultured neurons and in brain tissue; and understanding of the mechanism by which synaptic input controls a neuron's ability to modify its synapses.
